# Making Memories of Stressful Events: A Journey Along Epigenetic, Gene Transcription, and Signaling Pathways

**DOI:** 10.3389/fpsyt.2014.00005

**Published:** 2014-01-22

**Authors:** Johannes M. H. M. Reul

**Affiliations:** ^1^Neuro-Epigenetics Research Group, School of Clinical Sciences, University of Bristol, Bristol, UK

**Keywords:** stress, glucocorticoid hormone, epigenetic, MAPK, immediate-early gene, learning and memory, PTSD, anxiety

## Abstract

Strong psychologically stressful events are known to have a long-lasting impact on behavior. The consolidation of such, largely adaptive, behavioral responses to stressful events involves changes in gene expression in limbic brain regions such as the hippocampus and amygdala. However, the underlying molecular mechanisms were until recently unresolved. More than a decade ago, we started to investigate the role of glucocorticoid hormones in signaling and epigenetic mechanisms participating in the effects of stress on gene transcription in hippocampal neurons. We discovered a novel, rapid non-genomic mechanism in which glucocorticoids via glucocorticoid receptors facilitate signaling of the ERK-MAPK signaling pathway to the downstream nuclear kinases MSK1 and Elk-1 in dentate gyrus granule neurons. Activation of this signaling pathway results in serine10 (S10) phosphorylation and lysine14 (K14) acetylation at histone H3 (H3S10p-K14ac), leading to the induction of the immediate-early genes c-Fos and Egr-1. In addition, we found a role of the DNA methylation status of gene promoters. A series of studies showed that these molecular mechanisms play a critical role in the long-lasting consolidation of behavioral responses in the forced swim test and Morris water maze. Furthermore, an important role of GABA was found in controlling the epigenetic and gene transcriptional responses to psychological stress. Thus, psychologically stressful events evoke a long-term impact on behavior through changes in hippocampal function brought about by distinct glutamatergic and glucocorticoid-driven changes in epigenetic regulation of gene transcription, which are modulated by (local) GABAergic interneurons and limbic afferent inputs. These epigenetic processes may play an important role in the etiology of stress-related mental disorders such as major depressive and anxiety disorders like post-traumatic stress disorder.

## Introduction

The mnemonic function of the brain is one of its most important cognitive attributes. Making memories of events in our lives is vital in order to find, also quite literally, our way around in life. The formation of memories allows us to interact spatially, socially, and otherwise with our environment. As memories are built on representations from the environment, they are a pivotal part of how we adapt to changes in the environment thereby preparing us cognitively, emotionally, and physiologically should a similar situation occur in the future. We make particularly strong memories of traumatically stressful events in our lives. Most people cope well with such ordeals and stay healthy suggesting that they have adapted successfully. Some people however develop an anxiety disorder like post-traumatic stress disorder (PTSD), which seriously compromises their quality of life for a long time, often life-long. They are burdened by nightmares, reliving the incident recurrently, negative associations, mood swings, strong vegetative characteristics, and other debilitating symptoms. Clearly, strong memories have been encoded in these individuals but adaptation and coping mechanisms have failed. Until now, the question why 10–20% of the population develops a stress-related disorder after experiencing a traumatic, often life-threatening experience has remained unanswered. In order to answer this question, we need to obtain insight into how stress interacts with the emotional and cognitive processing of an event at the molecular level in the brain. Obtaining this knowledge is fundamental to answering the question about the neurobiological basis underlying the vulnerability for developing a stress-related mental disorder.

## Animal Models

The investigation of the effects of stress on learning and memory processes in the brain requires the use of animal models. The brain region which has received most attention is the hippocampus, a limbic brain structure, which is vital for the consolidation of contextual memories and plays a major role in coordinating the behavioral, autonomic, and neuroendocrine responses to stress. It has been shown in a number of studies that stress-induced glucocorticoid hormones (corticosterone in rats and mice) enhance the consolidation of memory formation in various hippocampus-dependent behavioral models including the contextual fear conditioning paradigm, the Morris water maze, and forced swimming-induced behavioral immobility (1–5). In fact, stress is inherent to these behavioral tests as a rodent is not keen on receiving an electric shock or being put in a water basin, this inadvertently leading to an enhanced secretion of glucocorticoid hormone from the adrenal gland (6).

**Figure 1:**
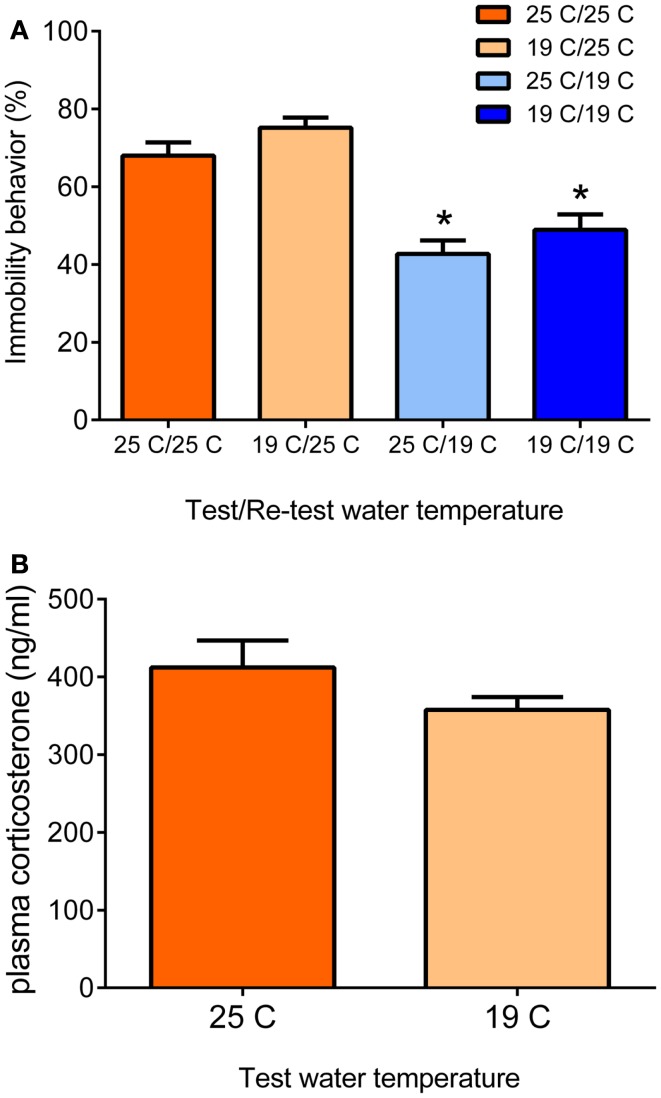
**Behavioral immobility response in the re-test depends on the water temperature during the re-test (A)**. Male Wistar rats were forced to swim for 15 min in 19 or 25°C-water (test). Twenty four hours later, they were forced to swim again for 5 min in 19 or 25°C-water (re-test). The data are presented as mean percentage immobility behavior during the 5-min re-test procedure ± standard error of the mean (SEM; *n* = 11–15) of different test/re-test water temperature conditions (see *x*-axis and legend). The data show that the immobility response in the re-test depends on the water temperature during the re-test and is independent of the water temperature at the time of the initial 15-min test. At 25°C re-test water temperature, the rats present the typical immobility response irrespective of the water temperature during the initial test. At 19°C re-test water temperature, the animals show significantly lower immobility scores, also irrespective of the water temperature during the initial test. Thus, in the Re-test, rats choose a behavioral strategy to cope with a challenge depending on conditions at the time of the challenge. The differential behavioral responses were not due to different responses in plasma glucocorticoid hormone **(B)**. Male Wistar rats were forced to swim for 15 min in 19 or 25°C-water and killed at 30 min after start of the forced swim procedure. Data are expressed as mean plasma hormone levels ± SEM (nanogram/milliliter; *n* = 9). Statistical analysis: **(A)** Two-way ANOVA: effect of test water temperature: *F*(1,49) = 3.718, *P* = 0.06 [Thus, there was a trend that if rats had swum at 19°C in the test, they would show a slightly higher immobility response in the re-test (see also Ref. (18)]. Effect of re-test water temperature: *F*(1,49) = 55.163, *P* < 0.0005. The interaction term was not statistically significant. **(B)** Student’s *t*-test: *P* > 0.05, not significant. Johannes M. H. M. Reul and Sabine Ulbricht conducted this study at the Max Planck Institute of Psychiatry in Munich, Germany, unpublished data.

An animal model often studied with regard to the role of glucocorticoid hormones in memory consolidation is the forced swim test. The strict dependency of the behavioral immobility response of glucocorticoid receptor (GR)-occupying levels of glucocorticoid hormone has been reported independently by two research groups in Utrecht, The Netherlands, and Melbourne, Australia (3, 5). The forced swim test is a rather straightforward test consisting basically of an (initial) test and a re-test. A rat or a mouse is put in a container (diameter < 30 cm) filled with water, usually 25°C, in which it cannot stand and from which it cannot escape. Usually, rats are left in for 15 min, mice often 10 min or shorter. After initial attempts of trying to escape by struggling or climbing movements vertically along the wall and horizontal swimming movements, the animal will acquire an immobile or floating posture. In the forced swim test’s classic design, the animals are re-introduced to the water container for 5 min, 24 h after the initial test (7, 8). In this 5-min re-test, the animals struggle or swim relatively little but show mainly (70–75% of the 5-min time-period) immobility/floating behavior. Recently, we reported that animals also show this enhanced immobility behavior when tested 4 weeks after the initial forced swim test (9). The rodent displays this behavioral immobility response in the re-test because it has learnt from the previous experience that attempting to escape is futile and thus conserving energy by floating or immobility behavior is the best strategy for survival (10–15). Moreover, the animal may remember that it was taken from the water at the time of the first test. Thus, the enhanced behavioral immobility behavior displayed in the re-test is an adaptive response, which is based on memories formed of the initial forced swim experience (13–15). The extent of the immobility behavior displayed depends on the conditions under which the test is conducted: in warmer water (e.g., 35°C), rats show more immobility behavior whereas they show less of this behavior in cold water (19°C) (16). Under the latter conditions, presumably the animals struggle and swim more to combat the vast body temperature loss (approximately 12°C within 15 min) due to the cold water (17). These observations underline that the animals adapt their behavior in response to the context of the test (see Figure 1).

The forced swim test has been often used as a psychopharmacological test for antidepressant drug screening. Acute treatment (up to three injections) of rats or mice with several (e.g., TCAs, SSRIs) but not all antidepressant drugs typically result in decreased immobility behavior and more struggling and swimming behavior in the test and the re-test (8). Notably, acute treatment with these drugs evokes increased extracellular concentrations of neurotransmitters like serotonin, noradrenalin, and/or dopamine in many forebrain structures; i.e., neurochemical changes, which under physiological conditions are associated with enhanced motor activity and arousal mechanisms. Accordingly, the increased struggling/swimming behavior observed after antidepressant drug administration may be explained by a disruption of the immobility behavior the animals would normally display in this test. Therefore, using the forced swim test as a pharmacological test for screening new drugs for their potential “antidepressant” activity has its limitations. Furthermore, it should be emphasized that this is a test for potential antidepressant drug action, not for depression. Hence, denoting rats or mice showing less struggling/swimming and more immobility behavior as being “depressed,” is inappropriate.

As mentioned, the behavioral immobility response observed in the re-test is critically dependent of glucocorticoid hormone action during or shortly after the initial test. Thus, glucocorticoids are needed for the acquisition and consolidation of memories associated with the stressful challenge (9, 18, 19). Behavioral responses in the re-test are impaired in adrenalectomized (ADX) rats, which can be rescued by administration of corticosterone or the synthetic glucocorticoids, dexamethasone and RU28362, but not by administration of the mineralocorticoid hormone aldosterone or the progestin progesterone (3, 5). Thus, of the two glucocorticoid-binding receptors in the brain, i.e., the mineralocorticoid receptor (MR) and the GR (20–22), the latter receptor type appeared to be the participating one. Furthermore, impairment of immobility behavior in the re-test (but not in the initial test) was observed if rats were pre-treated with the GR antagonist RU38486 but not with the MR antagonists, spironolactone or RU28318 (23, 24). As RU38486 also has anti-progestin activity, the role of progestins was further clarified. It was found that the effect of RU38486 on immobility behavior could be rescued with dexamethasone but not with the synthetic progestin, R5020 (promegestone) (23). To obtain insight into the identity of the neuroanatomical substrate of the GR-mediated glucocorticoid effect on the behavioral immobility response, De Kloet et al. infused RU38486 into the dentate gyrus (DG), parafascicular nucleus, or paraventricular nucleus of the hypothalamus before the initial swim test (23). They found a significantly impaired behavioral immobility response if the drug had been infused into the DG but not into any of the other nuclei (23). These findings indicated that GRs in the DG are particularly important for the consolidation of this behavioral response.

## Early Epigenetic Observations

The research pointing to a major role of glucocorticoid hormones in the forced swim test and other hippocampus-dependent tests was mainly conducted in the 1980s and early 1990s. For many years, it remained unclear how glucocorticoids are affecting these hippocampus-dependent behaviors. However, by the end of the 1990s, we made a serendipitous discovery: we found sparsely distributed granule neurons in the DG, which showed a speckled nuclear staining pattern for the chromatin-associated protein histone H3 phosphorylated at Serine10 (S10) and acetylated at Lysine14 (K14) (18). Nowadays, the code for this dual histone mark is H3S10p-K14ac. It turned out that under baseline conditions the number of immuno-positive neurons was very low but they increased considerably after psychologically stressful situations (known to be processed by the hippocampus) such as forced swimming, novelty, predator exposure, Morris water maze training, and fear conditioning (18, 25). Exposure to a cold environment or ether vapor, i.e., physical (non-hippocampal) stressors, was ineffective (18). Furthermore, we made an interesting observation that treating rats with the GR antagonists RU38486 or ORG34517 strongly inhibited the forced swimming-induced increase in H3S10p-K14ac-stained neurons in the DG (9, 18). ORG34517 is a rather novel GR antagonist, which has much less progesterone receptor antagonistic properties than RU38486 (26, 27). Based on *in vitro* work, it had been proposed that these epigenetic histone marks were involved in the opening of the chromatin structure rendering the hitherto silent genes located within this part of the chromatin accessible for transcription factors and other transcription-associated protein complexes and hence available for gene transcription (28–30). Thus, just after the turn of the millenium, our research had found evidence for stress- and glucocorticoid-sensitive histone modifications in DG neurons, which appeared to be related to transcriptional activation; a potentially interesting phenomenon but at the time not more than that. Moreover, at the time it was unclear whether this phenomenon had any bearing on the mechanisms underlying the behavioral immobility response.

Subsequent *in vitro* work of Mahadevan and colleagues in Oxford, UK, showed that the H3S10p-K14ac histone marks are associated with promoters of immediate-early genes (IEGs) like *fos* and *egr1* upon gene induction (31). We indeed found sparsely distributed c-Fos and Egr-1 immuno-positive neurons in DG of rats and mice, which increased in numbers after exposure to psychological stressors such forced swimming, novelty, and Morris water maze training (9, 18, 25, 32) (Carter et al., unpublished observation). Double immuno-fluorescence studies provided evidence that H3S10p-K14ac and c-Fos and Egr-1 protein co-localize in DG granule neurons (9, 25). Furthermore, pre-treatment of rats with the GR antagonist not only inhibited the forced swimming-evoked increase in H3S10p-K14ac in DG neurons but also strongly inhibited the stress effect on c-Fos and Egr-1 (9). Ultimate proof for a “physical” link between the H3S10p-K14 histone marks and c-Fos/Egr-1 was delivered by recent chromatin immuno-precipitation (ChIP) studies, which showed that these dual histone marks are present within the *fos* and *egr1* gene promoters of rats after forced swimming (9). Presently, ChIP studies on the H3S10p-K14ac and other histone marks in combination with next-generation Illumina sequencing are underway to make a genome-wide assessment of all genes associated with specific histone marks under baseline and stress conditions. So, it took a journey of more than 10 years for an interesting phenomenon to evolve to a potentially important epigenomic mechanism.

As early *in vitro* studies had linked the H3S10p-K14ac marks to IEG induction (31), it was thought that this link was universal, i.e., occurring in every *in vitro* and *in vivo* cell system. Our findings in numerous immunohistochemical and ChIP studies do not agree with this notion. It is well-known that induction of the IEGs c-Fos and Egr-1 occurs in a wide range of brain structures after exposure of experimental animals to various acute stressors. These brain structures include the whole neocortex including the prefrontal cortex, hypothalamic, thalamic, and amygdaloid nuclei, hippocampus (DG, CA1), and many pontine and brainstem nuclei (6, 33). The neuroanatomical immuno-localization of H3S10p-K14ac has turned out to be much more restricted with highest levels present in the DG (9, 18, 25, 32). Early studies found only very few H3S10p-K14ac-positive neurons outside the DG such as those in the neocortex, amygdala, and striatum. In more recent experiments, newer generations of antibodies and implementing immuno-staining techniques, which allow better antibody penetration find stronger staining among neurons in these brain regions (Carter et al., unpublished observations). However, remarkably, ChIP studies have found that only in the DG, H3S10p-K14ac is associated with the *fos* and *egr1* genes (9); thus, in other brain areas this dual histone mark is associated with other, as yet unknown genes. Apparently, in these brain areas histone acetylation and/or histone H3 K4 methylation is sufficient for IEG induction but this still needs to be investigated in detail. As the H3S10p-K14ac histone marks are thought to be associated with hitherto silent genes, several years ago we postulated that the c-Fos and Egr-1 gene promoters in the DG are in a different (condensed?) state than elsewhere in the brain (13, 34). In other words, the IEG promoters in this brain structure require the formation of the H3S10p-K14ac mark to open up (de-condense) to allow transcription factor binding and induction of gene transcription (14, 15).

## Finding the Path to the Chromatin

Despite these findings collected over the past 10 years, it remained a mystery how GRs were affecting epigenetic and gene transcriptional changes in dentate neurons in relation to the consolidation of stress-related memories and behavioral responses. It was clear from our studies that GRs in DG neurons play an important role in the phosphorylation of S10 and the acetylation of K14 and possibly other lysine residues at the n-terminal tail of histone H3 molecules within the *fos* and *egr1* gene promoters (9, 18). However, as GRs have no intrinsic kinase and histone acetyl-transferase activities, evidently the effects of this steroid receptor annex ligand-dependent transcription factor on H3S10p-K14ac formation were brought about in an indirect manner. Classically, GRs act through glucocorticoid-responsive elements (GREs) within promoter regions of glucocorticoid-responsive genes. Since the transient response in H3S10p-K14ac peaks at 30–60 min after stress, it was considered unlikely that GR-induced genes would be directly involved in modifying histone H3. This thought was strengthened by our observation that an injection of glucocorticoid hormone was ineffective in changing H3S10-K14ac in dentate neurons (25). Moreover, this observation excluded the possibility of a (fast) glucocorticoid effect via membrane-associated GRs. Thus, based on all available data, we postulated the involvement of (an) additional signaling pathway(s).

Since we regard the behavioral immobility response as a learned behavior, we postulated the participation of a pathway typically involved in learning and memory processes, i.e., the NMDA receptor-mediated ERK–MAPK pathway (ERK, extracellular signal-responsive kinase; MAPK, mitogen-activated protein kinase) (35–38). In a series of studies, we indeed found that the NMDA receptor antagonist MK801 and the MEK1/2 (MAPK ERK kinase 1/2) inhibitor SL327 strongly inhibited the forced swimming- and novelty-induced formation of H3S10p-K14ac and IEG expression (32). The effects of the MEK inhibitor indicated the involvement of a MAPK in the signaling pathway. Immunohistochemical analyses indeed showed the transient formation of phosphorylated ERK1/2 (pERK1/2) in DG neurons after forced swimming, but not of phosphorylated p38MAPK, underlining that there is specificity in the recruited MAPK pathway (9). pERK1/2 is however not a histone H3 kinase, thus an intermediary, histone H3 kinase needed to be sought. In collaboration with Dr. Simon Arthur (University of Dundee, UK), we studied mice with a double gene deletion for MSK1/2 (mitogen- and stress-activated kinase 1/2). MSK enzymes can be activated through phosphorylation by pERK1/2 (39). In MSK1/2 knock-out mice, we found virtually an absence of forced swimming-induced H3S10p-K14ac formation and c-Fos induction in dentate granule neurons and a severe impairment of the behavioral immobility response in the re-test (32). Furthermore, Dr. David Sweatt and colleagues at Baylor College (USA) reported in MSK1 gene deleted mice an impaired performance in the Morris water maze and in the contextual fear conditioning paradigm (40). In rats, we found a transient increase in the number of sparsely distributed pMSK1/2 immuno-stained granule neurons in the DG after forced swimming (9). There was no staining of the phosphorylated form of the MSK-related kinases RSK1/2 (pRSK1/2; phosphorylated ribosomal S6 kinase 1/2), which are also substrates of pERK1/2, again underlining specificity. Elk-1 (Ets-like protein kinase) plays an important role in the induction of IEGs like c-Fos and Egr-1 (41–43). *In vitro* work has shown that, upon phosphorylation, for instance by pERK1/2, pElk-1 can bind to the Elk-1 binding site within the SRE(s) [serum response element(s)] and exert trans-activational influences within the *fos* or *egr1* gene promoters (42, 43). pElk-1 has been shown to fulfill these effects through recruitment of the histone acetyl-transferase p300, which after phosphorylation by pElk-1, acetylates nearby histone H3 tails at K14 and other lysine positions (42, 44). After forced swimming, we found an increase in pElk-1 stained DG neurons that time wise paralleled the responses in pERK1/2 and pMSK1/2 (9). Thus, it appears that the phosphorylation and acetylation of histone H3 occur in a coordinated fashion.

Using pharmacological tools and a gene deletion model, we had identified a pathway of interlinked signaling partners that convey extracellular signals (glucocorticoid hormones, glutamate) triggered by environmental challenges (e.g., forced swimming) to the epigenome resulting in gene transcriptional changes in DG neurons. However, the sparse distribution pattern of pERK1/2, pMSK1/2, pElk-1, H3S10p-K14ac, c-Fos, and Egr-1 immuno-positive granule neurons within the DG prompted the critical postulate that, in order to build a true signaling cascade, all signaling molecules needed to co-exist in the same neurons. GRs and NMDA receptors are ubiquitous in the DG. Elaborate double immuno-fluorescence analyses provided the final evidence that, within sparsely distributed DG granule neurons, forced swimming and other psychologically stressful challenges indeed activate GRs and the NMDA/ERK1/2/MSK1/2-Elk-1 signaling pathways resulting in H3S10p-K14ac formation and IEG induction (9).

From a functional perspective, it was of critical importance to show that the NMDA/ERK1/2/MSK1/2-Elk-1 signaling pathway is essential for the forced swimming-induced behavioral immobility response. Using the aforementioned pharmacological and gene deletion approaches, we found that any intervention of this signaling cascade resulted in an impairment of the behavioral response (9, 18, 32). Reports of other investigators have provided strong evidence for an involvement of the NMDA receptor, ERK-MAPK signaling, and MSK1 receptors in the consolidation of spatial and emotional memories associated with the Morris water maze paradigm and contextual fear conditioning (36, 40, 45) (Carter et al., unpublished).

## Cross-Talk of Signaling Pathways: A Novel Mechanism of Glucocorticoid Action

**Figure 2:**
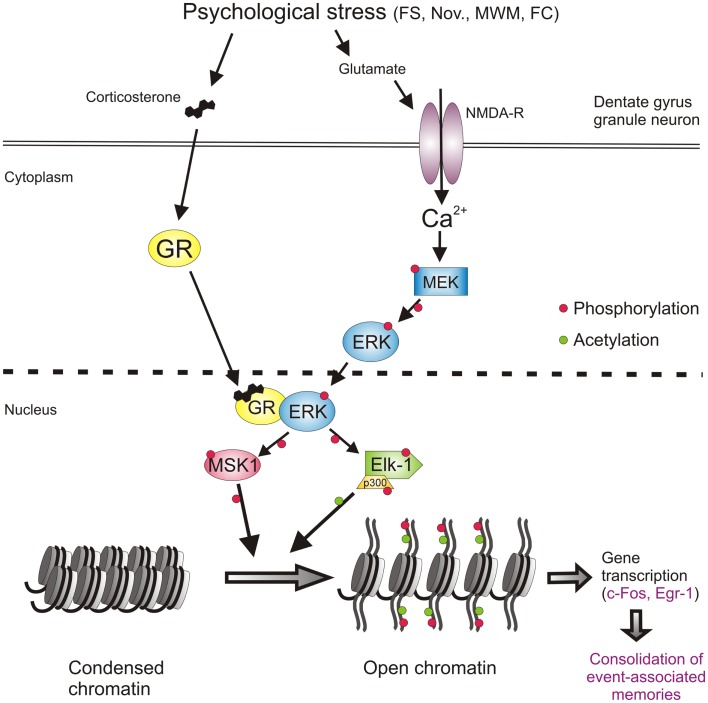
**Psychological stress-activated signaling pathways in dentate gyrus granule neurons driving epigenetic modifications underlying induction of gene transcription and the consolidation of behavioral responses and memory formation**. Psychological stress evokes the concomitant activation of the GR and NMDAR-ERK-MAPK pathways. The concomitant activation of ERK1/2 and GR and their subsequent physical interaction facilitates the ability of pERK1/2 to phosphorylate MSK1/2 and Elk-1. Activation of these nuclear kinases results in the phosphorylation and acetylation of histone H3 (H3S10p-K14ac), which drives chromatin remodeling thereby allowing the gene transcription of IEGs like *c-fos, egr1*, and many other genes. The induction of gene transcription is critical for the consolidation of memory formation associated with the endured event. See text for references of studies supporting this concept. FS, forced swimming; Nov., novelty exposure; MWM, Morris water maze training; FC, contextual fear conditioning.

Thus, at this stage, the involvement of a second major signaling pathway, the NMDA/ERK1/2/MSK1/2-Elk-1 pathway, in the forced swimming-induced immobility response, and possibly Morris water maze behavior and contextual fear conditioning, had been resolved. The identification of this second pathway however as yet did not provide insight into the mechanism of action of the GRs on behavior. The outstanding question had remained whether GRs affect behavior through interaction with the ERK MAPK pathway. To address this question, the role of GR activity was investigated in the forced swimming-induced ERK1/2, MSK1/2, and Elk-1 phosphorylation by pre-treating rats with the GR antagonist RU38486 before the stress challenge. The GR antagonist did not affect the stress effect on pERK1/2 but strongly inhibited the formation of pMSK1/2 and pElk-1 in the dentate neurons (9) suggesting that the drug was acting downstream from pERK1/2 and more so that pERK1/2 required activated GRs to phosphorylate MSK1/2 and Elk-1. This is consistent with the earlier notion that these glucocorticoid effects do not involve membrane-associated GRs. Follow-up co-immuno-precipitation studies showed that GR and pERK1/2 indeed undergo physical interactions after forced swimming to facilitate the generation of pMSK1/2 and pElk-1 [Figure 2; (9)]. Thus, our data showed evidence for a novel mechanism in which GRs act like a scaffold to facilitate the phosphorylation of MSK1/2 and Elk-1 by pERK1/2 (Figure 2). These effects of GRs take place within 15 min after start of forced swimming, thus not as fast as the reported membrane GR-mediated effects (46) but much quicker than the classical genomic effects of this corticosteroid receptor (30–60 min). Thus, we have uncovered a novel non-genomic mode of action of glucocorticoids, which is taking place in the immediate-early time domain after stress (9).

## Anxiety Status as Determinant Factor: Role of GABA

The emotional or anxiety status plays a profound role in the impact of psychologically stressful events on physiological, behavioral, and cognitive responses. Accordingly, it is thought that more anxious individuals make stronger memories of stressful events than less anxious individuals. Since many years, great research efforts are vested into the identification of risk factors for the development of PTSD after traumatic experiences. For instance, physical abuse during childhood, thus previous trauma, has been found to be a risk factor for the development of PTSD in Vietnam War veterans (47, 48). More recent work suggests that people who are more anxious have an increased risk for developing PTSD after a traumatic event (49). We were interested to which degree the anxiety status would affect molecular mechanisms in DG neurons known to be involved in memory formation of stressful events. We wanted to explore the role of anxiety by applying three different approaches.

The first approach made use of the rodent’s innate fear of open spaces and bright light. We had observed that the mild psychological stressor novelty exposure resulted in an enhanced formation of H3S10p-K14ac and induction of c-Fos in the rat DG (25). The novelty paradigm entailed that the group-housed rat would be individually placed in a cage with new bedding in an unfamiliar room illuminated with a rather bright light source (450–500 lx). For rodents, this is a stressful condition as they instinctively fear predation in well-lit open spaces. We observed that exposing rats to a new cage under light conditions akin those in the holding room (100 lx) led to hardly any rise in H3S10p-K14ac and c-Fos in dentate neurons. However, increasing the light intensity (up to 500 lx) progressively resulted in stronger novelty-induced histone and IEG responses in these neurons (25). Thus, increasing the anxiogenicity of the stressful condition enhanced the epigenetic and gene transcriptional responses in the dentate granule neurons.

Secondly, we addressed the factor anxiety by adopting a pharmacological approach by using the anxiolytic or anxiogenic properties of certain GABAergic drugs. A well-known GABA-related anxiolytic drug is the benzodiazepine Lorazepam. Pre-treatment of rats with an anxiolytic dose of Lorazepam (high doses are sedative) resulted in a complete blockade of the H3S10p-K14ac and c-Fos responses to novelty (50). In contrast, application of the partial inverse agonist FG7142 produced strongly augmented novelty-evoked responses in these histone and IEG marks (50). FG7142 has been shown to increase the excitability of neurons in other parts of the brain to airjet and predator exposure (51, 52) and is known to be strongly anxiogenic in rodents and humans (53–56). The drug lowers GABA-A function and clearly has actions opposite those of Lorazepam. In aggregate, it is clear that GABA plays a major role in the modulating effect of anxiety on responsiveness of dentate neurons to stressful events.

The third approach concerns the application of the voluntary exercise paradigm. Research over the past 15 years has shown that allowing rats and mice access to a running wheel has major beneficial effects for their health and wellbeing. Rats and mice run voluntarily in a wheel during their active period of the day reaching on average a distance of approximately 4 and 6 km per day, respectively (57–59). If this type of voluntary exercise was allowed for several weeks, it proved to have remarkable beneficial effects on a broad range of cellular, physiological, and behavioral processes including neurogenesis in the DG (60–63), the central control of HPA axis responses to psychologically stressful events (57–59, 64), on sleep quality (increased slow wave sleep) (65), and on cognitive, impulsive, and emotional behavior (60, 66). Using the elevated plus-maze, dark–light box, and the novel cage paradigm, long-term exercise was found to have strongly diminishing effects on anxiety levels in both rats and mice (66). Although still somewhat controversial, regular exercise has been shown to have beneficial effects in anxious and depressed patients (67, 68). Therefore, in many countries exercise is presently prescribed to such patients as a co-treatment in addition to the classical pharmacological and behavioral therapies. We investigated whether exercise would impact on stress-induced behavior. We found that exercised rats showed remarkably different behavior in the novel cage paradigm than sedentary animals (69). When sedentary rats are placed alone in a new cage (lights: 500 lx), they explore the novel environment for the full 30 min the test lasts. However, exercised rats explored the new cage for only 10–15 min after which they laid down to rest or sleep, i.e., the normal behavior a rat displays during the daytime (69). Thus, apparently exercised animals much quicker reach the conclusion that the new environment is safe, which corresponds with their lower anxiety level and possibly enhanced cognition. The reduced anxiety levels in the exercised rats may be due to changes in their GABAergic system. We reported that in addition to distinct changes in the expression of GABA-A receptor subunits (e.g., the extra-synaptic receptor associated delta and alpha-5 subunits), long-term exercise resulted in an increased gene transcription of the GABA synthesizing enzyme GAD67 (70). Furthermore, our recent preliminary findings suggest that GABA synthesis capacity is increased in the DG of exercised rats (Kersanté et al., unpublished observations).

We investigated changes in ERK-MAPK signaling and c-Fos expression in the DG after long-term voluntary exercise. We found that 4 weeks of wheel running resulted in a significant attenuation of the forced swimming-induced increases in the pERK1/2, pMSK1/2, and c-Fos (Collins et al., unpublished observations). Thus, exercised rats show reduced ERK-MAPK and IEG responses to forced swim stress, which may be a consequence of the enhanced GABAergic inhibitory tone in the DG of these animals (50).

Together, this work strongly supports the notion that the anxiety state and the state of the GABAergic system play a pivotal role in the responsiveness of DG granule neurons to psychological stress. In molecular terms, this responsiveness is translated into the likelihood of initiation of ERK-MAPK signaling, epigenetic changes, and induction of (IEG) gene transcription. The findings also suggest that anxiety acts upon these molecular mechanisms through modulation of the GABAergic tone arising from local interneurons in the DG. The GABAergic tone is regulated locally by adjustments in GABAergic synthesis and release capacity, GABA-A receptor subunit composition as well as through afferent inputs from other regions of the brain (see below).

The observation that particularly fearsome and emotional events impact strongly on the extent of activation of dentate granule neurons may underlie the well-known phenomenon that such events are typically very strongly stored into memory, often life-long. In terms of behavioral adaptation and from an evolutionary perspective this makes great sense. Keeping track of potential predators, conspecific enemies, dangerous places, and other threats is crucial to stay safe and avoid violence and predation. Evidently, the level of anxiety awareness and GABAergic control need to be tightly regulated to remain healthy and safe. Hyper-anxiety/low GABAergic control may be profoundly debilitating (humans: social isolation, incapability; animals: social isolation, starvation) whereas low anxiety/high GABAergic control may be dangerous to the individual (humans: carelessness; accident-prone, sensation-seeking behavior: injuries, death; animals: predation). In addition to these state-dependent variables affecting the health condition of humans and animals, repeated challenges leading to chronic stress as well as gravely traumatic life events (e.g., rape, abuse, extreme violence and horror, like in war situations, and other near-death experiences) can lead to depressive and anxiety disorders like PTSD (71, 72) possibly through damage and/or dysfunction of the DG and other parts of the hippocampal formation.

## Role of Afferent Input to the Dentate Gyrus

In addition to local (GABAergic) mechanisms regulating DG excitability also extra-hippocampal, afferent input to this hippocampal region is of principal importance. It has been established that information flow through the hippocampus is modulated by various afferent inputs from subcortical regions of the brain including the septum (73), locus coeruleus (74), raphe nuclei (75, 76), amygdala (77, 78), and the supra-mammillary area (SMA) in the hypothalamus (79–82).

### The supra-mammillary area, a hypothalamic region involved in integrating cognitive and emotional behavior

The hippocampal formation receives substantial afferent projections from the SMA, which are channeled through the fimbria–fornix. This input has a strong influence on hippocampal theta rhythms and is therefore thought to play an important role in hippocampus-dependent cognitive functions and emotional behavior. Lesions of the mammillary area, including the SMA, have been reported to result in impaired spatial learning and memory in several behavioral tasks including the water maze (83–86). The neuroanatomy of the SMA–hippocampus connection is complex and has been investigated for many years. Although the neuroanatomy has not been fully clarified, the projections seem to consist of glutamatergic and GABAergic fibers, which predominantly innervate the DG and to a lesser extent the CA2/CA3a region of the hippocampus (87–89). Physiologically, the main effect of SMA stimulation is the facilitation of perforant path-elicited population spikes in the DG (80, 90–92). This facilitation has been thought to result from a disinhibition mechanism due to GABAergic SMA–DG afferents inhibiting local dentate GABAergic interneurons (80). Such afferents on DG interneurons have however not been found and it seems that virtually all SMA-DG afferent fibers project to granule neurons in the DG (88). Presently, supported by anatomical studies (88, 93–95), it is thought that the SMA potentiates population spikes evoked by perforant path stimulation in the DG via direct excitatory glutamatergic synaptic neurotransmission upon granule neurons (95). Yet, Nakanishi et al. reported that SMA-evoked facilitation of perforant path stimulated EPSP spikes in DG was blocked by the GABA-A blocker picrotoxin leaving the possibility open for a disinhibitory role of GABAergic interneurons in granule neuron excitability (92). It seems that, using both excitatory and inhibitory afferent inputs, the SMA plays a pivotal role in facilitating information flow in the DG in a behavior-dependent manner (96).

### Interplay of the SMA and the amygdala

Evidence has been accumulating that a role of the SMA in controlling information flow in the hippocampus also affects the influence of the amygdala on hippocampus function. Since many years, it has been known that the amygdala plays a pivotal role in hippocampus-mediated learning and memory processes associated with emotion. Although neuroanatomically a link between the amygdala and DG has not been clarified yet, physiological research has provided ample evidence for the existence of such link. It has been shown that lesioning or functionally inhibiting the basolateral amygdala attenuates long-term potentiation (LTP) in the DG (97, 98). Stimulation of this amygdala region facilitates perforant path-DG synaptic responses (78). Furthermore, high-frequency stimulation of the medial amygdala evokes a long-lasting potentiation of perforant path-DG population spikes (99). McGaugh et al. reported functional neuroanatomical evidence that injection of NMDA into the amygdala evokes the induction of c-Fos in the DG (100, 101).

Thus, afferent projections from the SMA and the amygdala to the DG play an important facilitatory role in merging the influence of anxiety/emotionality with the multimodular sensory information flow through the DG. This physiological mechanism appears to be instrumental in facilitating the formation of memories of emotionally charged life events. At the DG cellular level, the coordinated inputs from the SMA and amygdala resulting in an enhanced excitability of dentate neurons may translate into an enhanced likelihood of NMDAR-mediated excitation of dentate granule neurons resulting downstream into activation of signaling, epigenetic, and gene transcriptional changes known to be required for the consolidation of memory formation. Clearly, research is required to provide substance to this notion.

## Significance of H3S10p-K14ac Formation for Induction of Gene Transcription

The induction of the IEGs, c-Fos and Egr-1, in DG granule neurons after psychological stressors such as forced swimming and novelty requires the formation of dual histone mark H3S10p-K14ac (9, 25, 32). However, although *in vitro* research suggests that the association of this dual histone mark with IEG gene induction is a general phenomenon, in the brain *in vivo* this is not the case. Using ChIP, we found that the dual histone mark is only associated with the c-Fos and Egr-1 promoter region in the DG but not in the neocortex (9). In view of evidence that the H3S10p-K14ac histone mark is associated with the opening of dormant genes (28, 30, 31), we have concluded that under baseline conditions the c-Fos and Egr-1 genes and possibly many other genes in the DG are in a different, i.e., condensed, state and require the formation of the dual histone mark in their gene promoters for de-condensation and gene transcription (13–15, 34, 102).

Presently, it is unknown why IEG induction in DG neurons, as opposed to other neurons in the brain, is critically dependent of formation of H3S10p-K14ac. Possibly, as part of the physiological, sparse activation scheme applied in the DG, gene induction in dentate neurons is required to be strictly regulated, apparently to safeguard that after an environmental challenge only a few percent of DG neurons are responding. Thus, dentate neuron activation and function is controlled at several levels including the tonic GABAergic control and other afferent (SMA, amygdala) control at the cellular level, the NMDA receptor-mediated Ca^2+^/ERK-MAPK requirement at the signaling level and the requirement of H3-S10pK14ac-driven chromatin remodeling at the molecular level. In addition to the dual histone mark, our recent work suggests that also the DNA methylation status of distinct CpGs within the *fos* and *egr1* gene promoters play a critical role in the transcriptional activity at these genes after stress (Saunderson et al., in preparation). This additional epigenetic mechanism adds another level of molecular control of IEG expression in DG neurons.

Recently, we postulated that these histone modifications and the opening of the chromatin structure are needed to provide transcription factors like CREB access to their DNA binding sites (14, 15). CREB is a well-known trans-activator of c-Fos and Egr-1 gene transcription. Our concept attempts to explain the observation that, although CREB phosphorylation occurs ubiquitously in the DG after stressful challenges like forced swimming (6), c-Fos and Egr-1 are only expressed in those DG granule neurons in which H3S10p-K14ac has been generated (9, 25, 32).

To obtain deeper insight into the functional implications of the H3S10p-K14ac mark in dentate neurons, we have started ChIP studies in combination with Illumina next-generation sequencing (ChIP-Seq). This genome-wide screen will deliver detailed knowledge about the identity of all genes specifically associated with the dual histone mark, other histone marks as well as those genes, which show binding of distinct transcription factors within their gene promoters. Thus, in other words, this state-of-the-art approach will inform us about which genes in the activated dentate neurons are specifically involved in adaptive changes in these neurons after a stressful challenge. This information will help to obtain insight into the functional changes occurring in these neurons and furthermore will assist in the elucidation of the functional properties of as yet unknown genes.

## Outlook

As mentioned at the beginning of this text, adaptation to stressful events in our lives, which includes the formation of memories of such events, is of critical importance to maintain health and well-being. Anxiety disorders like PTSD and major depressive disorder are thought to be the consequence of disruptions and impairments in this adaptive process. The likelihood of developing this kind of mental disorders is higher in individuals who have been subject to early life abuse or neglect, or have endured massive, acute traumatic events or chronic psychological stress. The observation that “only” 10–20% of people develop a mental disorder under such conditions suggests the involvement of genetic factors as well as “phenotypic” factors (e.g., age, lifestyle, socio-economic status), which determine an individuals’ resilience to stress.

The DG plays a pivotal role in the encoding of incoming sensory and other information from the entorhinal cortex involving pattern separation (103, 104) that enables the CA3 to utilize this information for further processing and integration, which is key to the formation of event-associated memories. This NMDAR-dependent process in sparse dentate granule neurons evokes long-term molecular and cellular changes in these neurons, which subserve long-lasting changes in functional properties of these cells. Our work has helped to gain insight into the signaling, epigenetic, and gene transcriptional mechanisms evoked in these dentate neurons after a psychologically stressful challenge. Previously, we have suggested that these mechanisms act like a molecular switch allowing hippocampal information processing and thereby the consolidation of memories associated with the challenge (13). The generation of the dual histone mark H3S10p-K14ac, driven by concomitant activation of the GR and NMDAR/ERK1/2/MSK1-Elk-1 pathways, plays a central role in kick-starting gene transcription required for long-term changes in neuron function. This concept provides the framework for the identification and investigation of the gene products involved beyond the IEGs c-Fos and Egr-1. Furthermore, it invites investigating the role of afferent input from subcortical brain structures in the regulation of DG granule neuron function at the molecular level. Together, this research opens up the opportunity for preclinical and clinical studies on the pathophysiological significance of the participating genes and thereby hopefully for drug development to combat stress-related mental disorders.

## Conflict of Interest Statement

The author declares that the research was conducted in the absence of any commercial or financial relationships that could be construed as a potential conflict of interest.
